# Unlocking the Potential of Secondary Data for Public Health Research: Retrospective Study With a Novel Clinical Platform

**DOI:** 10.2196/51563

**Published:** 2024-10-01

**Authors:** Christopher Gundler, Karl Gottfried, Alexander Johannes Wiederhold, Maximilian Ataian, Marcus Wurlitzer, Jan Erik Gewehr, Frank Ückert

**Affiliations:** 1 Institute for Applied Medical Informatics University Medical Center Hamburg-Eppendorf Hamburg Germany; 2 Research Data Facility University Medical Center Hamburg-Eppendorf Hamburg Germany

**Keywords:** secondary use, hypothesis testing, research platform, clinical data, Parkinson disease, data, health-related research, health data, electronic health record, EHR, tremor

## Abstract

**Background:**

Clinical routine data derived from university hospitals hold immense value for health-related research on large cohorts. However, using secondary data for hypothesis testing necessitates adherence to scientific, legal (such as the General Data Protection Regulation, federal and state protection legislations), technical, and administrative requirements. This process is intricate, time-consuming, and susceptible to errors.

**Objective:**

This study aims to develop a platform that enables clinicians to use current real-world data for testing research and evaluate advantages and limitations at a large university medical center (542,944 patients in 2022).

**Methods:**

We identified requirements from clinical practitioners, conceptualized and implemented a platform based on the existing components, and assessed its applicability in clinical reality quantitatively and qualitatively.

**Results:**

The proposed platform was established at the University Medical Center Hamburg-Eppendorf and made 639 forms encompassing 10,629 data elements accessible to all resident scientists and clinicians. Every day, the number of patients rises, and parts of their electronic health records are made accessible through the platform. Qualitatively, we were able to conduct a retrospective analysis of Parkinson disease over 777 patients, where we provide additional evidence for a significantly higher proportion of action tremors in patients with rest tremors (340/777, 43.8%) compared with those without rest tremors (255/777, 32.8%), as determined by a chi-square test (*P*<.001). Quantitatively, our findings demonstrate increased user engagement within the last 90 days, underscoring clinicians’ increasing adoption of the platform in their regular research activities. Notably, the platform facilitated the retrieval of clinical data from 600,000 patients, emphasizing its substantial added value.

**Conclusions:**

This study demonstrates the feasibility of simplifying the use of clinical data to enhance exploration and sustainability in scientific research. The proposed platform emerges as a potential technological and legal framework for other medical centers, providing them with the means to unlock untapped potential within their routine data.

## Introduction

In recent years, there has been a growing interest in using clinical routine data, especially electronic medical records, for research [1]. Known as secondary data use, this practice is significantly influenced by legislative actions such as the Health Information Technology for Economic and Clinical Health Act in the United States of America and publicly founded initiatives like the Medical Informatics Initiative (MII) in Germany [2,3]. University hospitals now serve as central hubs in bridging the gap between research and patient care. Achieving connectivity between previously isolated data silos necessitates adherence to detailed standards, such as the custom FHIR (Fast Healthcare Interoperability Resources) profile “Kerndatensatz” in Germany and effective communication among diverse stakeholders within national health systems [4]. Despite the inherent complexity, these concerted efforts are expected to establish a new foundation for evidence-based science.

Despite the advantages of adopting a state-wide approach to secondary data use, we contend that certain research could be more effectively conducted at the local level within individual hospitals. Specifically, we have identified 2 critical use cases where hypothesis testing on unmapped raw data is essential for advancing evidence-based medicine:

First, to verify hypotheses derived from clinical practice on a larger database, clinicians should be able to validate their experiences easily through data-driven investigations. These studies may involve data elements not comprehensively covered by standardized data sets. In addition, expecting clinicians to create intricate mappings between used data elements and state-wide standards may prove ineffective.

Second, the replication of existing publications to assess their generalizability must always consider the local context. Conducting public health research, for example on large cohorts of patients with Parkinson disease (PD), might miss important external factors [5,6]. Accordingly, testing external validity on a schema applied in practice rather than one developed for collaboration appears more appropriate.

Beyond the clinical perspective, local solutions may better accommodate regional or local legal requirements, as many collaborative standardizations tend to converge on the “smallest common divisor” between partners. Consequently, the implementation of complementary systems for secondary data analysis at both the local and global levels is deemed appropriate.

Developments in recent years have led to the emergence of research platforms that enable the analysis of clinical data in compliance with data protection guidelines. Notable examples include EPOCH and ePRISM (IP-ITT Corporation) [7], KETOS (Friedrich-Alexander University Erlangen-Nürnberg) [8], and Medical-Blocks (University of Bern) [9]. These platforms provide environments that allow clinical scientists to train and deploy statistical models. However, their primary focus is on translating these models back into clinical practice rather than testing hypotheses through the secondary use of data. In addition, works such as EHR4CR (Electronic Health Records for Clinical Research) [10] have implemented infrastructures that enable the use of clinical data across multiple European sites in a secure and privacy-preserving manner without focusing on the subsequent analysis. Given our knowledge, a platform for hypothesis testing on routine data has not been implemented and evaluated in clinical reality.

This paper addresses this gap in research by introducing and evaluating a novel platform explicitly designed for hypothesis testing on clinical routine data. Starting by collecting the requirements of clinicians, we strive to design and implement a modular and, consequently, reusable platform. Similar to other states, the federal law of Hamburg permits pseudonymized retrospective data analysis without patient consent given specific guarantees regarding data protection. The platform ensures those guarantees in accordance with all European and German laws and is directly integrated into the technical infrastructure of the University Medical Center Hamburg-Eppendorf (UKE). The evaluation process encompasses quantitative assessment, exemplified by the replication of a public health finding in the context of PD, and qualitative evaluation through the examination of clinicians’ use in real-world clinical scenarios. This dual-pronged evaluation strategy aims to judge both the quantitative efficacy and practical use of the proposed platform in clinical reality.

## Methods

### Technical Considerations

For developing the platform, we examined the challenges of using routine hospital data for hypothesis testing through extensive communication with the different business divisions of the UKE, like the infrastructure department, division for information technology, research data facility, data protection officers, and internal boards. Informed by the project meetings and discussions with clinicians as later users, we identified and prioritized 4 critical process components necessitating optimization.

#### Defining Appropriate Hypotheses

Precise hypothesis formulation relies on a thorough understanding of metadata within the clinical information system. For researchers, the accessibility of relevant data fields may not be immediately evident. Challenges arise from both nontechnical limitations and the opacity of data type and structure. Filtering cohorts based on specific criteria may yield statistically inappropriate sizes, and requested data may be inadequately recorded [11]. The feasibility of research ideas is thus not guaranteed, necessitating extensive consultation with data integration experts for hypothesis refinement.

#### Obtaining Data From the Infrastructure

Efficient storage and retrieval of routine hospital data are crucial for medical treatment and research. Hospitals use diverse IT architectures, often a mix of specialized systems with proprietary data structures and nonstandardized file formats. Access and control vary widely, from centralized systems to more federated approaches led by individual clinics. Clinicians aiming to test hypotheses face challenges in accessing required documentation, understanding these structures, and communicating with the responsible data manager.

#### Analysis of the Hypotheses

To facilitate hypothesis tests, clinicians expressed a need for a comprehensive and heterogeneous array of tools, encompassing table-based software and standard scripting languages like Python (Python Software Foundation) or R (R Foundation for Statistical Computing). Established research data management platforms, such as Kaggle (Google) [12], Paperspace Gradient (DigitalOcean) [13], Colab (Google) [14], or CodaLab (Microsoft Research) [15], provide ideal support for efficient data analysis: An integrated and simplified development environment, a separate space for data analysis with access to high-performance computing, and the ability to communicate and collaborate with other users of the research community. Rather than developing a novel solution, leveraging a platform that accommodates diverse analysis methods appears to be a pragmatic approach.

#### Reuse of Established Components

Based upon the preliminary work of the MII and the existing research landscape, the following tools were explored as relevant in the context of our work.

#### Data Integration

Data integration centers (DICs) enable the cross-site and cross-institutional use of digital health data from patient care and biomedical research in Germany [2,3,16]. All DICs are located at university medical sites and have access to routinely collected patient data. To this end, they build up interoperable databases with quality-assured and internationally harmonized data (based on HL7 [Health Level Seven International] FHIR) and metadata. These are made available in anonymized form through trustees. DICs make an important contribution to the development of a research-orientated infrastructure for the German health care system. The first use cases using the functionalities are already in operation [17]. These functionalities are reusable and valuable for our work. For further details, we refer to the literature regarding the MII [2,3].

#### Data Usage Considerations

European, national, and local laws govern the use of sensitive routine data. Those projects necessitate ethical approval and explicit consent, a crucial yet burdensome process for both researchers and ethics committee members. As the legislators have already identified the need for simplification, we were able to use §12 of the “Hamburgisches Landeskrankenhausgesetz” [18]. This statute permits pseudonymized retrospective data analysis without patient consent, allowing us to forego consent-based data usage. With approval from the ethics committee for hypothesis testing, the board and the individual researcher might focus more on the research question rather than time-consuming bureaucratic processes. Without this general approval for hypothesis testing, researchers would normally not be able to query the data without extensive knowledge regarding the infrastructure and the law. Furthermore, the UKE has established an independent trust center, which is largely autonomous in legal terms. This center uses suitable pseudonymization techniques to safeguard patient data identity.

#### Metadata Processing

The processing of metadata is crucial in the context of data harmonization with multiple data sources, as intended in this project. Metadata repositories (MDRs) enable the structuring of data for the technical extract, transform, and load (ETL) process. They are also applications that make the syntax and semantics of the data understandable for the end user. Both attributes are relevant in our context. Numerous systems have already been tested and evaluated in use [15-17]. In this case, we prefer an MDR that is a further development of the already used Samply.MDR [19], an ISO/IEC 11179-based metadata repository built on a graph-based backend, making the MDR applicable to many hierarchical data structures.

### Quantitative and Qualitative Analysis

In the evaluation of the proposed tool, a dual assessment was conducted, encompassing a qualitative analysis of its suitability for replicating a public health-focused study and a quantitative examination of clinicians’ usage behavior within the hospital.

#### Qualitative Analysis: Hypothesis Testing

The capabilities of the proposed platform for testing scientific hypotheses appear to be valuable for replicating studies in other cohorts. Comparable to existing publications [8], we applied the platform to underscore its efficacy in promoting sound scientific practices and for examining the generalizability of findings regarding the circumstances present at a specific hospital.

Due to its notable clinical implications [20,21], we chose PD as a neurodegenerative disorder of interest for which routine data may provide helpful insights. The International Parkinson and Movement Disorder Society (MDS) developed a scoring system to measure the severity of PD motor symptoms. This movement test is called the Unified Parkinson’s Disease Rating Scale (UPDRS) and is widely used in clinical routine [22]. While postural, kinetic, and isometric tremor are subcategories of action tremor, the isometric tremor is difficult to measure in routine clinical settings and is not routinely assessed [23,24]. Nonetheless, the exact relationship between these distinct types of tremors remains incompletely understood.

Motivated by the findings of Gupta et al [25], our objective is to validate their proposed correlation between rest tremor and action tremor in patients with PD [26]. Consequently, we aim to replicate their observation of a significantly higher prevalence of action tremor in individuals also experiencing resting tremor.

By leveraging the proposed platform, we gained access to routine data, expanding beyond the use of public data sets used in the original study: The Parkinson Progression Marker Initiative (PPMI) [27], the Fox Investigation for New Discovery of Biomarkers (BioFIND) [28], and the Parkinson’s Disease Biomarkers Program (PDBP) [29] data sets are 3 distinct clinical oriented, observational studies collecting relevant disease-specific data from patients with PD. The PPMI study focuses on early-stage patients with PD who have recently been diagnosed and are not yet receiving dopaminergic treatment. In contrast, the BioFIND and PDBP studies encompass patients at varying stages of PD, ranging from moderate to advanced and early to advanced, respectively. Consequently, the PPMI data set exclusively includes patients in the medication-off state, while the latter 2 data sets include patients in both the medication-off and medication-on states.

As the first step of the analysis, we identified those forms within the clinical information system used to store classifications according to the MDS-UPRDS. The platform facilitated the selection of a well-defined cohort, ensuring precise inclusion criteria for the data query. Accordingly, we included all patients with the designated *ICD-10-GM* (*International Statistical Classification of Diseases and Related Health Problems, Tenth Revision, German Modification*) code for Parkinson disease (G20). Furthermore, we limited our cohort to admissions that occurred between February 24, 2018, and February 24, 2023. Although age limitations can be applied, we did not impose any restrictions for the presented cohort. Leveraging the aforementioned criteria, we executed the query and retrieved all corresponding records stored in the system.

#### Quantitative Analysis

For the quantitative analysis, we focus on performance indicators critical for assessing the relevance of our platform in clinical reality. The practical use of the platform is measured with the cumulative probability distribution and the absolute number of requests after the initialization of the platform. The waiting times are critical for user experience, which is expected of the researcher’s experience when they receive the requested data.

### Ethical Considerations

Based on the proposed pipeline for pseudonymization and data security, the ethics committee of the Hamburg chamber of physicians agreed on approval for all hypothesis tests conducted through the platform (2022-100891-BO-ff).

## Results

### Technical Realization Within Clinical Reality

**Figure 1:**
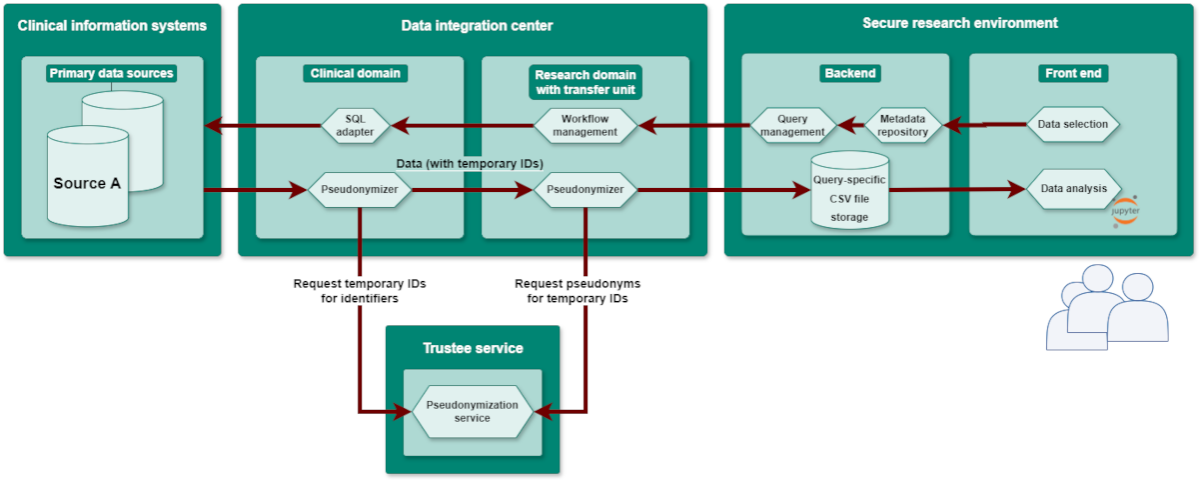
Components and data processing architecture of the platform. CSV: comma-separated value; SQL: Structured Query Language.

The central implementation detail of the platform is the usage of the established systems through strategic interfacing within the DIC. Notably, it circumvents the integration phase by directly querying the databases of clinical systems. The platform’s backend is incorporated into the DIC’s clinical and research domains, using the central trustee service for pseudonymization and the transfer unit for managing workflows and facilitating data delivery to researchers. The underlying architecture is constructed using standardized web technologies, specifically HTTPS and REST (Representational State Transfer). These procedural steps are fully automated, furnishing transparent feedback on the ongoing progress (Figure 1).

The data architecture is organized into 3 primary sections: clinical information systems, data integration center, and secure research environment. A researcher initiates hypothesis testing using the web application in the front end of the secure research environment. Data selection for this process is facilitated through a catalog of data elements supplied by the metadata repository. The data integration center, which is divided into 2 domains, the clinical domain, and the research domain, is responsible for converting the researcher’s query into potentially multiple SQL (Structured Query Language) commands to retrieve data from the clinical information system’s database. The output is exported as a CSV (comma-separated values) file and subjected to the pseudonymization triangle, where identifiers like medical record numbers and visit numbers are replaced with temporary IDs by the clinical domain before transmitting the data set to the research domain. The trustee service ensures that data from various sources are assigned consistent pseudonyms, maintaining the integrity of the hypothesis testing context. Subsequently, the pseudonymized data is stored in the query-specific CSV file storage within the backend of the secure research environment. Researchers have access to these data sets for further analysis and can use analytical tools such as Jupyter notebooks, which are available on the front end.

Text data or images that contain sensitive information are not exported. Subsequently, the research domain decodes temporary IDs into definitive pseudonyms and stores the data set for subsequent researcher access. Both the clinical and research domains obtain temporary IDs and pseudonyms from the trustee service, configured to issue unique ones for each identifier type (eg, medical record number or visit number). The linkage between original IDs and pseudonyms remains confidential to both domains; solely, the trustee service retains this information throughout the project’s duration. After this process, the resulting file is automatically downloaded into a separate network designed for research and stored on a file system accessible only to the requesting clinician.

Using a widely recognized solution for both clinicians and data scientists, we incorporated JupyterLab, a prominent open-source web-based development environment, as the principal front end for ensuing data analyses. As a result, the proposed platform empowers users to leverage a diverse array of tools and libraries on the 639 forms encompassing 10,629 data elements that we have made accessible from clinical routine.

### Qualitative Results: Example of a Hypothesis Testing

For the qualitative assessment, all patients with the designated *ICD-10-GM* code G20 and admissions that occurred between February 24, 2018, and February 24, 2023, were included. The majority of patients underwent multiple assessments during their hospital visit. We only considered the first assessment to ensure independent samples and discarded subsequent assessments. This was necessary since we were mainly interested in the patient cohort with any of the 3 basic tremor types rather than the overall occurrence of all tremors assessed at any given time point. The decision to choose the first assessment was made since not every subject was assessed more than once, but always at the beginning of their hospitalization, thereby ensuring uniformity in tremor severity assessments shortly after admission. Afterward, we derived the subtypes described in the original work by considering the MDS-UPDRS items 3.17, 3.15, and 3.16 as surrogates for rest tremor, postural tremor of the hands, and kinetic tremor of the hands, respectively. As a result, we were able to include 777 patients in our qualitative assessment.

Table 1 presents the prevalence of the primary tremor types and the association between rest tremor and action tremor. The table includes 4 distinct data sets, with the first 3 data sets obtained from Gupta et al [25] and the fourth data set corresponding to our analysis conducted using the proposed tool (UKE). The provided values for rest tremor, postural tremor, and kinetic tremor represent the count of patients with PD exhibiting each respective tremor type while at rest, while holding their hands stretched out, or during a finger-to-nose maneuver, respectively. The severity rating for each tremor type is equal to or above 1, as outlined in the MDS-UPDRS guideline. The aggregated values presented in the table were derived following the published protocol.

**Table 1:** Comparison of the tremor subtypes and their occurrences within the cohorts reported by Gupta et al [25]. In addition, the last column shows the obtained results from routine data based on the proposed platform.

	PPMI^a^ (N=423), n (%)	BioFIND^b^ (N=118), n (%)	PDBP^c^ (N=874), n (%)	UKE^d^ (N=777), n (%)
Rest tremor	290 (68.6)	75 (63.6)	459 (52)	340 (43.8)
Pure rest tremor	87 (20.6)	15 (12.7)	104 (11.8)	57 (7.3)
Action tremor	156 (36.9)	46 (39)	316 (35.8)	255 (32.8)
Pure action tremor	40 (9.5)	10 (8.5)	87 (9.9)	76 (9.8)
Postural tremor	223 (52.7)	69 (58.5)	412 (46.7)	416 (53.5)
Pure postural tremor	18 (4.3)	8 (6.8)	31 (3.5)	72 (9.3)
Kinetic tremor	217 (51.3)	61 (51.7)	463 (52.5)	301 (38.7)
Pure kinetic tremor	23 (5.4)	6 (5.1)	86 (9.8)	31 (4)
No tremor	52 (12.3)	19 (16.1)	211 (23.9)	258 (33.2)
Any tremor	317 (87.7)	99 (83.9)	663 (75.2)	519 (66.8)
All tremor	116 (27.4)	36 (30.5)	229 (26)	179 (23)

^a^PPMI: Parkinson Progression Marker Initiative.

^b^BioFIND: Fox Investigation for New Discovery of Biomarkers.

^c^PDBP: Parkinson’s Disease Biomarkers Program.

^d^UKE: University Medical Center Hamburg-Eppendorf.

Our results represent a cohort of patients with PD irrespective of any dopaminergic treatment since many patients lack information regarding medication status due to the subsequent addition of this data field into the clinical information system. Through our data analysis, we observed a prevalence of 43.8% (340/777) for rest tremors and 7.3% (57/777) for pure rest tremors within the cohort. In contrast, the prevalence of total action tremor was 32.8% (255/777), with a corresponding occurrence of 9.8% (76/777) for pure action tremor. The incidences of postural tremor and pure postural tremor were found to be 53.5% (416/777) and 9.3% (72/777), respectively. We identified a prevalence of 38.7% (301/777) for kinetic tremor and 4.0% (31/777) for pure kinetic tremor. Finally, we calculated the occurrence of patients exhibiting all 3 tremor types simultaneously, the absence of any tremor, and the presence of at least 1 tremor type, resulting in proportions of 23.0% (179/777), 33.2% (258/777), and 66.8% (519/777), respectively. These relative figures closely resemble the reported values from the original authors. Importantly, we also observed a significantly higher proportion of action tremors in patients with rest tremors (43.8%) compared with those without rest tremors (32.8%), as determined by a chi-square test (*P*<.001).

Our analysis of routine data has yielded additional evidence that aligns with the published findings, suggesting that action tremor may be part of a broader tremor syndrome observed in PD. This discovery emphasizes the need for a more dynamic approach to tremor classification, considering the progressive worsening of rest tremor severity over time [30] and its potential association with the occurrence of action tremor. Specifically, our data set corroborates the previous findings by Gupta et al [25], which propose a relationship between rest tremor and the emergence of action tremor. The data we have obtained further suggests that action tremor may represent a manifestation of the underlying basal ganglia disease, highlighting the potential requirement for additional neuroimaging studies to elucidate this connection.

### Quantitative Results

In the realm of quantitative results, we focus on performance indicators critical for assessing the relevance of our platform within the clinical reality. To that end, we compiled a comprehensive list of successful queries executed using our proposed tool before October 30, 2023. Subsequently, we exclude queries carried out by members of the development teams, as they were primarily intended for debugging purposes.

**Figure 2:**
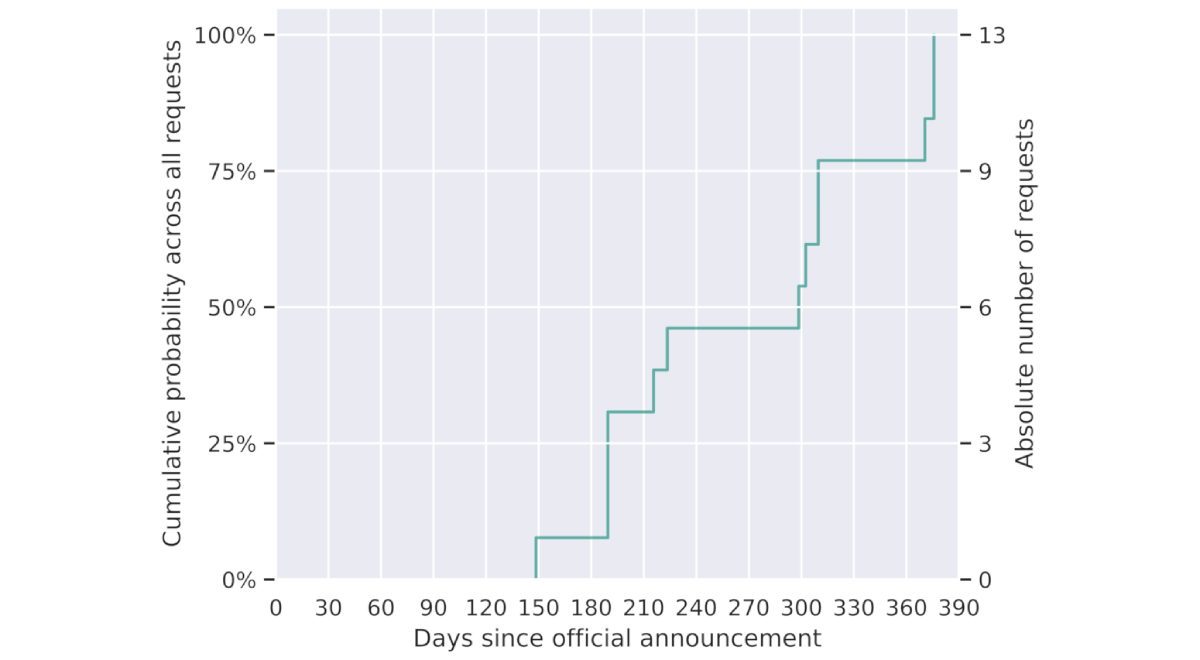
Cumulative probability distribution of researcher-initiated queries over time, starting from the public announcement of the platform, as extracted from the platform logs.

Figure 2 illustrates the cumulative probability distribution of all incorporated queries across the temporal dimension. A conspicuous observation is the initial absence of queries in the early phase, signifying a notable delay in the adoption of the tool by clinical researchers, spanning nearly 6 months. The discernible rightward shift indicates an increasing interest among researchers following an initial habituation period. Nevertheless, the following data points reveal a marked acceleration in query use, with over 50% of the total queries executed within the most recent 3-month period.

Our platform offers the unique advantage of accessing multiple systems integral to clinical care. However, it’s important to note that these platforms are not optimized for the specific nature of the queries in question. Substantial delays in data retrieval could significantly impede the quality of research conducted using our tool. Consequently, we analyzed to evaluate the waiting times experienced by clinical researchers before they received the requested data.

**Figure 3:**
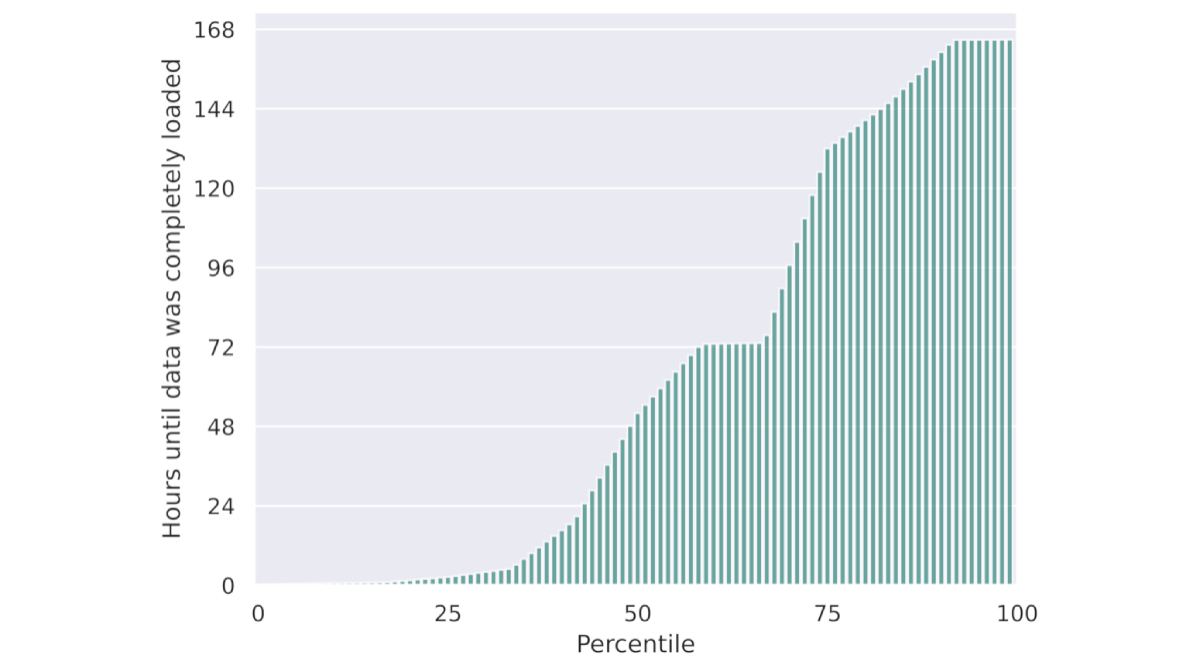
The percentiles illustrate all queries conducted by researchers until the availability of data for analysis extracted from the platform logs.

Figure 3 displays the distribution of the time it took for the queried data to become available to the researcher. The plot reveals a notable range of waiting times. While more than 50% of all requests were processed within a time frame of 50 hours, the longest queries extended to nearly a week. The pronounced initial ascent up to the median highlights the prompt reception of a substantial proportion of data despite the existence of instances where requests experience prolonged processing durations.

## Discussion

### Principal Findings

The development of a novel data platform at the University Medical Center Hamburg-Eppendorf for hypothesis testing on current clinical routine data according to all European and German data protection laws is accepted and used by clinicians. Accordingly, designing, implementing, and establishing a streamlined process for conducting hypothesis testing in public health by using secondary data appears possible. The initial version presented in this study involves the development of an analytical platform with a data protection-compliant infrastructure and a comprehensive ethical mandate, which will be extended with respect to semantic and syntactic interoperability found in the literature. This innovation has culminated in the establishment of a tool in clinical reality, which occupies a unique niche within the national health care landscape.

Given the illustrative use case, our findings indicate that routine data can facilitate the creation of data sets on scales comparable to prospective studies within significantly shorter time frames than those. This observation carries profound implications for diverse hospital roles: Patients gain transparency and trust in research processes, as the platform serves as a reliable authority for consent, enhancing confidence in the hospital’s practices. Clinicians find empirical support for hypothesis testing, aiding in evidence-based decision-making and simplifying time-consuming replication studies. The Data Protection Officer benefits from automated queries, reducing project-related risk management burdens and minimizing infringement risks through a secure, tested architecture. Research data infrastructure experts receive structured support for handling researchers’ queries. Finally, the hospital itself benefits from the efficient use of routine clinical data, offering potential cost savings, increased efficiency, and enhanced competitiveness.

Beyond the scope of our study, there is a discernible increase in interest in the tool within clinical reality. Over the last 90 days, the number of successful queries has doubled, and in total, clinical data from 600,000 patients or 1.6 million cases were retrieved from the platform. Although the absolute figures remain constrained, there is evident adoption by clinical researchers, indicating active use of the new tool for their hypothesis tests.

### Limitations

Our investigation underscores that the long execution times of queries on general-purpose databases in clinical systems, which are not inherently designed for the queries executed by the research platform, can limit the interactivity of researchers with the clinical data. Similar systems in other hospitals may likely face comparable issues. The complexity of supporting various query formulations through SQL query adapters further complicates optimization, often resulting in less efficient query statements compared with those that are meticulously crafted by hand. To enhance our platform and achieve shorter execution times, further development is essential. By now, we established a time limitation for queries, terminating excessively large ones. In the future, we plan to use strategies such as horizontally scaling the data sources, using alternative data stores or data caches, or using FHIR search or the Clinical Query Language as the query mechanism instead of traditional SQL [31].

Furthermore, our observations indicate a lack of universal intuitiveness among clinical users in our hospital regarding the Jupyter Notebooks used for analysis. Despite the formulation of data queries, the execution of analyses experienced a notable decline. The participation of clinicians in platform design underscores a potential gap in data literacy among individual physicians. To mitigate this, we advocate for an additional reduction in the entry barrier through the introduction of user-friendly, broadly applicable dashboards and visualizations tailored to each data query.

An additional aspect that holds potential for enhancing usability in the future is the ability to share access to analysis spaces. This feature would enable users with limited statistical expertise to invite statistical or biomedical experts into their analysis space, gradually receiving support throughout the analysis process. By allowing collaborative access, inexperienced users can benefit from the guidance and assistance of domain experts, facilitating their learning and development in statistical analysis. Accordingly, this feature is currently under development.

### Conclusions

With the presented research platform, we were able to establish a valuable tool for hypothesis testing and secondary use of clinical data. By automating the retrieval process of pseudonymized clinical data and providing a clear legal framework, the platform contributes to the facilitation of the research process. The practical usability of the platform was demonstrated through the replication of a scientific study using the example of PD, confirming the validity of the concept. In further development stages and through the integration of additional clinical data sources, we aim to continuously increase the quantity of data and the usability of the platform. In the long term, through further modularization and standardization, the platform should be made usable for additional national and European sites, significantly facilitating the secondary use of clinical data.

## Abbreviations

BioFIND: Fox Investigation for New Discovery of Biomarkers
CSV: comma-separated values
DIC: data integration center
EHR4CR: Electronic Health Records for Clinical Research
ETL: Extract, Transform, Load
FHIR: Fast Healthcare Interoperability Resources
HL7: Health Level Seven International
*ICD-10-GM*: *International Statistical Classification of Diseases and Related Health Problems, Tenth Revision, German Modification*
MDR: Metadata Repository
MDS: International Parkinson and Movement Disorder Society
MII: Medical Informatics Initiative
PD: Parkinson disease
PDBP: Parkinson’s Disease Biomarkers Program
PPMI: Parkinson Progression Marker Initiative
REST: Representational State Transfer
SQL: Structured Query Language
UKE: University Medical Center Hamburg-Eppendorf
UPDRS: Unified Parkinson’s Disease Rating Scale

## Data Availability

The anonymized data sets regarding the usage behavior analyzed during this study are available in the ZFDM repository [32]. The clinical data sets are not publicly available due to the missing legal foundation for the export of routine data but are available from the corresponding author on reasonable request and given permission from the required access committees.
